# Predictive and prognostic value of ZEB1 protein expression in breast cancer patients with neoadjuvant chemotherapy

**DOI:** 10.1186/s12935-019-0793-2

**Published:** 2019-03-29

**Authors:** Ziping Wu, Lei Zhang, Shuguang Xu, Yanping Lin, Wenjin Yin, Jinglu Lu, Rui Sha, Xiaonan Sheng, Liheng Zhou, Jinsong Lu

**Affiliations:** Department of Breast Surgery, Renji Hospital, School of Medicine, Shanghai Jiao Tong University, No. 160 Pujian Road, Pudong New District, Shanghai, 200127 People’s Republic of China

**Keywords:** Breast cancer, Neoadjuvant chemotherapy, ZEB1, Predictive, Prognostic

## Abstract

**Background:**

Zinc finger E-box binding homeobox 1 (ZEB1) is a molecule involved in the progression of epithelial-to-mesenchymal transition (EMT) in various kinds of cancers. Here, we aimed to determine whether the expression of the ZEB1 protein is related to the response of patients to neoadjuvant therapy as well as their survival outcome.

**Methods:**

Immunohistochemistry (IHC) was performed on paraffin-embedded tumor samples from core needle biopsy before neoadjuvant therapy (NAT). Univariate and multivariate logistic regression analyses were used to analyze the associations between the protein expression of ZEB1 and the pathological complete response (pCR) outcome. Kaplan–Meier plots and log-rank tests were used to compare disease-free survival (DFS) between groups. A Cox proportional hazards model was used to calculate the adjusted hazard ratio (HR) with a 95% confidential interval (95% CI).

**Results:**

A total of 75 patients were included in the IHC test. High ZEB1 protein expression was associated with a low pCR rate in both univariate (OR = 0.260, 95% CI 0.082–0.829, *p *= 0.023) and multivariate (OR = 0.074, 95% CI 0.011–0.475, *p *= 0.006) logistic regression analyses. High ZEB1 protein expression was also associated with a short DFS according to both the log-rank test (*p *= 0.023) and Cox proportional hazard model (HR = 9.025, 95% CI 1.024–79.519, *p *= 0.048). In hormone receptor positive (HorR-positive) patients, high ZEB1 protein expression was also associated with a lower pCR (OR = 0.054, 95% CI 0.007–0.422, *p *= 0.005) and a poorer DFS (HR = 10.516, 95% CI 1.171–94.435, *p *= 0.036) compared with low ZEB1 protein expression. In HER2-overexpressing patients, ZEB1 protein expression was also associated with poor survival (*p *= 0.042).

**Conclusions:**

Our results showed that high ZEB1 protein expression was a negative predictive marker of pCR and DFS in neoadjuvant therapy in breast cancer patients and in HorR-positive and HER2-overexpressing subgroups.

*Trial registration* NCT, NCT02199418. Registered 24 July 2014—Retrospectively registered, https://clinicaltrials.gov/ct2/show/NCT02199418?term=NCT02199418&rank=1. NCT, NCT 02221999. Registered 21 August 2014—Retrospectively registered, https://clinicaltrials.gov/ct2/show/NCT02221999?term=NCT02221999&rank=1

## Background

Neoadjuvant therapy (NAT) is routinely administered in the treatment of breast cancer, and the response rates range from 15 to 25% with traditional chemotherapy regimens [1], and the number spiked to 40–58% with combined chemotherapy and targeted drugs [2–4]. NAT is used not only for locally advanced patients with a large tumor burden, providing surgery opportunities and breast-conserving opportunities, but also to predict patients’ responsiveness to treatment at an early stage. Pathological complete response (pCR) refers to the status when no tumor cell residues or only ductal carcinoma in situ remains in the surgical specimens after preoperative treatment. A number of large-scale clinical trials have confirmed that patients who reached pCR in NAT presented with a much better prognosis than patients without pCR [2, 5]. However, not all patients achieve pCR results using the same NAT treatment. Therefore, predicting a patient’s response to NAT at an early stage and determining the predictive factor of pCR have recently become hot issues.

Zinc finger E-box binding homeobox 1 (ZEB1) is a transcription factor physiologically involved in cell differentiation and tissue development [6–9]. Recently, a growing amount of evidence suggested the role of ZEB1 in oncogenesis by driving the process of epithelial-to-mesenchymal transition (EMT) [10, 11]. Despite aberrant ZEB1 expression in various kinds of human cancers, including breast cancer [12–15], it was also recognized that this protein might play a pivotal role in therapeutic resistance [16, 17]. For instance, temozolomide is a standard chemotherapeutic drug used in glioblastoma treatment. Inhibition of ZEB1 expression reduces both invasion and resistance to temozolomide [16]. Similarly, the level of ZEB1 expression was also found to be correlated with resistance to gemcitabine and 5-fluorouracil [16, 17] in pancreatic cancer. However, whether ZEB1 expression is involved in the response to neoadjuvant chemotherapy in breast cancer has yet to be determined.

In this study, we aimed to explore the correlation between ZEB1 protein expression and neoadjuvant chemotherapy sensitivity in patients from our weekly paclitaxel- and cisplatin-based neoadjuvant trial in breast cancer. Considering the role of ZEB1 in oncogenesis, EMT progression and drug resistance, we hypothesized that the expression of ZEB1 is correlated with a poor response to neoadjuvant chemotherapy and poor prognosis for patients with breast cancer.

## Materials and methods

### Patient cohort

Breast cancer patients from two paclitaxel- and cisplatin-based neoadjuvant clinical trials were included in this study. The two trials were separately registered on the ClinicalTrials.gov website as SHPD001 (NCT02199418) and SHPD002 (NCT02221999). SHPD001 and SHPD002 were reviewed and approved by the independent ethical committee and institutional review board of the Renji Hospital, Shanghai Jiao Tong University. All patients provided written informed consent.

Women aged ≥ 18 years old with histologically confirmed locally advanced invasive breast cancer were included. For all patients, paclitaxel 80 mg/m^2^ was given weekly on day 1 for 16 weeks, and cisplatin 25 mg/m^2^ was given weekly on days 1, 8 and 15 every 28 days for 4 cycles as the NAT regimen. For HER2-positive patients in SHPD001, trastuzumab was recommended concurrently. All HER2-positive patients in SHPD002 received concurrent trastuzumab. For hormone receptor-positive patients in SHPD002, endocrine therapy of aromatase inhibitor with or without gonadotropin releasing hormone agonist was randomized according to their menstrual status. Planned surgery was given sequentially after NAT. All procedures performed in studies involving human participants were in accordance with the ethical standards of the institutional and/or national research committee and with the 1964 Helsinki declaration and its later amendments or comparable ethical standards.

The primary outcome of SHPD001 and SHPD002 was pCR, which was defined as the absence of invasive tumors in the breast and axillary lymph node samples, which were removed at the time of surgery.

### Immunohistochemistry (IHC)

Estrogen receptor (ER), progesterone receptor (PR), Ki-67, HER2 and ZEB1 were performed on paraffin-embedded tumor samples from biopsy. ER, PR, HER2 and Ki-67 were detected using rabbit monoclonal antibodies SP1, EE2, 4B5 (F. Hoffmann-La Roche Ltd., Switzerland) and MIBI (Leica Biosystems Newcastle Ltd., UK). ZEB1 was detected using the goat anti-ZEB1 (E-20) monoclonal antibody (Santa Cruz Biotechnology, Dallas, TX, USA).

ER and PR positivity was defined as more than 1% positive nuclear staining, and Ki-67 levels were recorded as a continuous value. HER2 assessments were conducted according to the American Society of Clinical Oncology (ASCO)/College of American Pathologists (CAP) recommendations 2013 [18]. ZEB1 evaluation was conducted according to the following criteria. The percentage of positively stained tumor cells was graded on a four-point scale [19] as follows: 1 (less than 20% positive cells), 2 (20–40% positive cells), 3 (50–70% positive cells) or 4 (greater than 70% positive cells). Staining intensity was subjectively assessed only in malignant epithelial tissue according to a four-point scoring system comprising 1 (−), 2 (+), 3 (++) or 4 (+++). The final ZEB1 results were scored by multiplying the percentage of positively stained tumor cells by staining intensity, dividing ZEB1 expression into negative and/or weak staining (≤ 6) and strong staining (> 6).

### Statistical analysis

Correlations between ZEB1 expression and other clinicopathological characteristics were tested using the Chi squared test. Univariate and multivariate logistic regression tests were used to analyze the associations between different ZEB1 expression levels and pCR outcome with calculated odds ratio (OR). Disease-free survival (DFS) was used for survival analysis. DFS was defined as the time from breast surgery to the first disease relapse, including one of the following events: distant disease metastasis, recurrence of ipsilateral locoregional invasive disease, contralateral breast cancer and death of any cause. The survival curve was derived from the Kaplan–Meier method; the log-rank test was used to compare the survival rate. A Cox proportional hazards model was used to calculate the adjusted hazard ratio (HR) with a 95% confidential interval (95% CI). Patient age, tumor size, ER expression, PR expression, HER2 expression and Ki-67 level were adjusted.

Statistical results were considered significant with a p value < 0.05. All statistical analyses were carried out using STATA statistics SE 14 (Stata Corp LP, College Station TX).

## Results

### Baseline characteristics

**Fig. 1:**
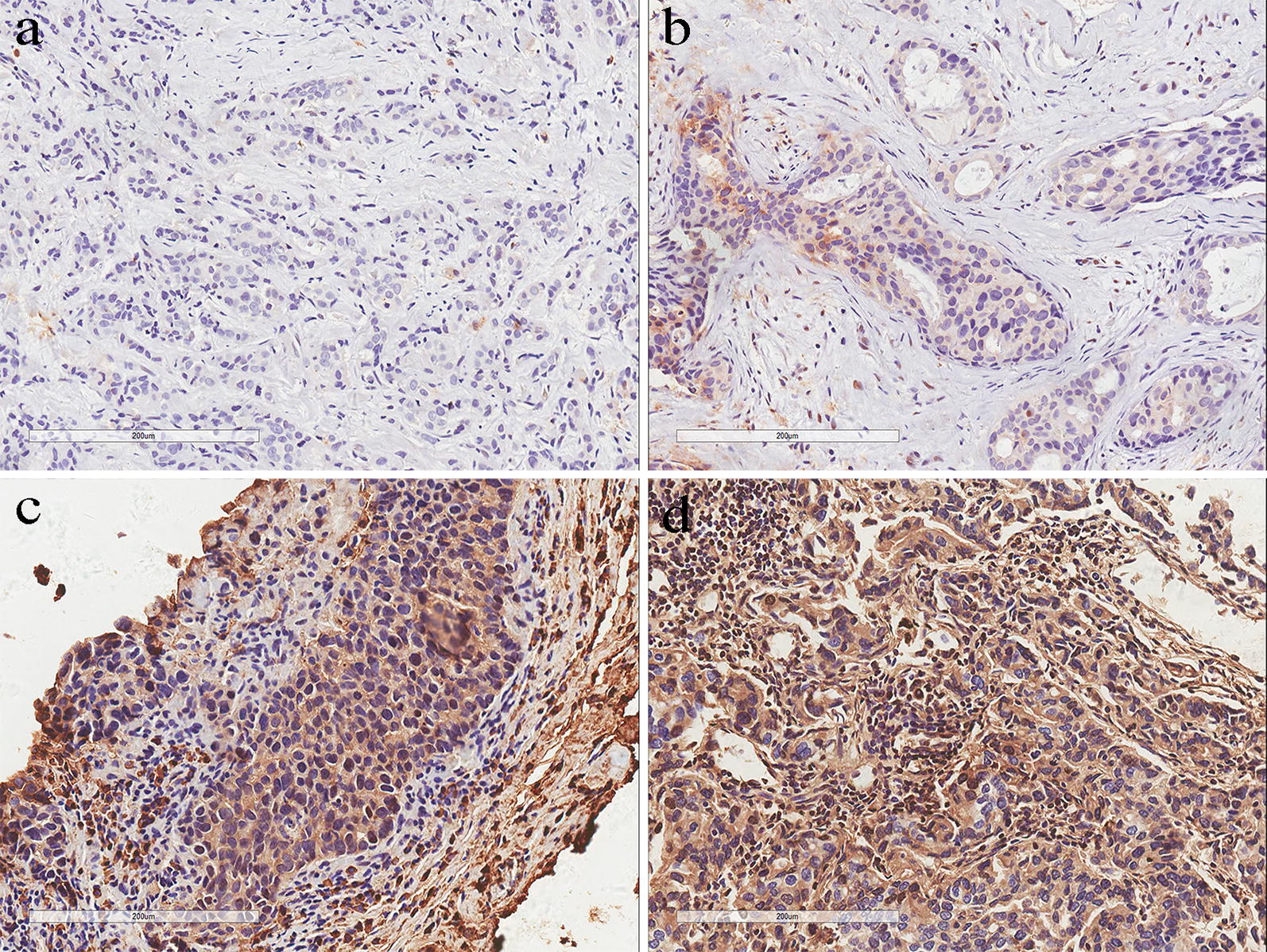
Different ZEB1 immunochemistry staining. **a** Weak ZEB1 expression with a staining score of 1 (≤ 6); **b** weak ZEB1 expression with a staining score of 6 (≤ 6); **c** strong ZEB1 expression with a staining score of 8 (> 6); **d** strong ZEB1 expression with a staining score of 12 (> 6)

A total of 75 patients were included in the analyses. Fifty-three percent of patients had a tumor larger than 5 cm, 52.0% of patients had a Ki-67 level of more than 30%, 49.3% of patients were younger than 50 years old, and 24.0% of patients received pCR after NAT. Representative tissue stainings of ZEB1 are presented in Fig. 1. ZEB1 expression was related to patient age and menopausal status in this cohort of patients (Table 1).

**Table 1:** Association between ZEB1 expression and clinicopathological factors in breast cancer

Variables	Number (%)	Number (%)	*p* value
	ZEB1 negative and weak expression	ZEB1 strong expression	
Age			
> 50	23 (30.6)	14 (18.7)	*0.015*
≤ 50	13 (17.3)	25 (33.3)	
Tumor size (cm)			
> 5	19 (16.8)	23 (30.7)	0.308
≤ 5	17 (22.7)	16 (21.3)	
ER status			
Positive	25 (33.3)	32 (42.7)	0.202
Negative	11 (14.7)	7 (9.3)	
PR status			
Positive	32 (42.7)	32 (42.7)	0.403
Negative	4 (5.3)	7 (9.3)	
Ki-67 status (%)			
> 30	17 (23.7)	22 (29.3)	0.426
≤ 30	19 (25.3)	17 (23.7)	
HER2			
Positive	13 (17.3)	15 (20.0)	0.833
Negative	23 (30.6)	24 (32.0)	
Lymph node positive			
> 0	15 (20.0)	23 (30.6)	0.134
≤ 0	21 (28.0)	16 (21.3)	
Menopausal status			
Premenopausal	23 (30.6)	14 (18.7)	*0.015*
Postmenopausal	13 (17.3)	25 (33.3)	

Siginificant *p* values are given in italic (*p* < 0.05)

The *χ*^2^ test was used for comparison between groups

### ZEB1 expression and pCR

**Fig. 2:**
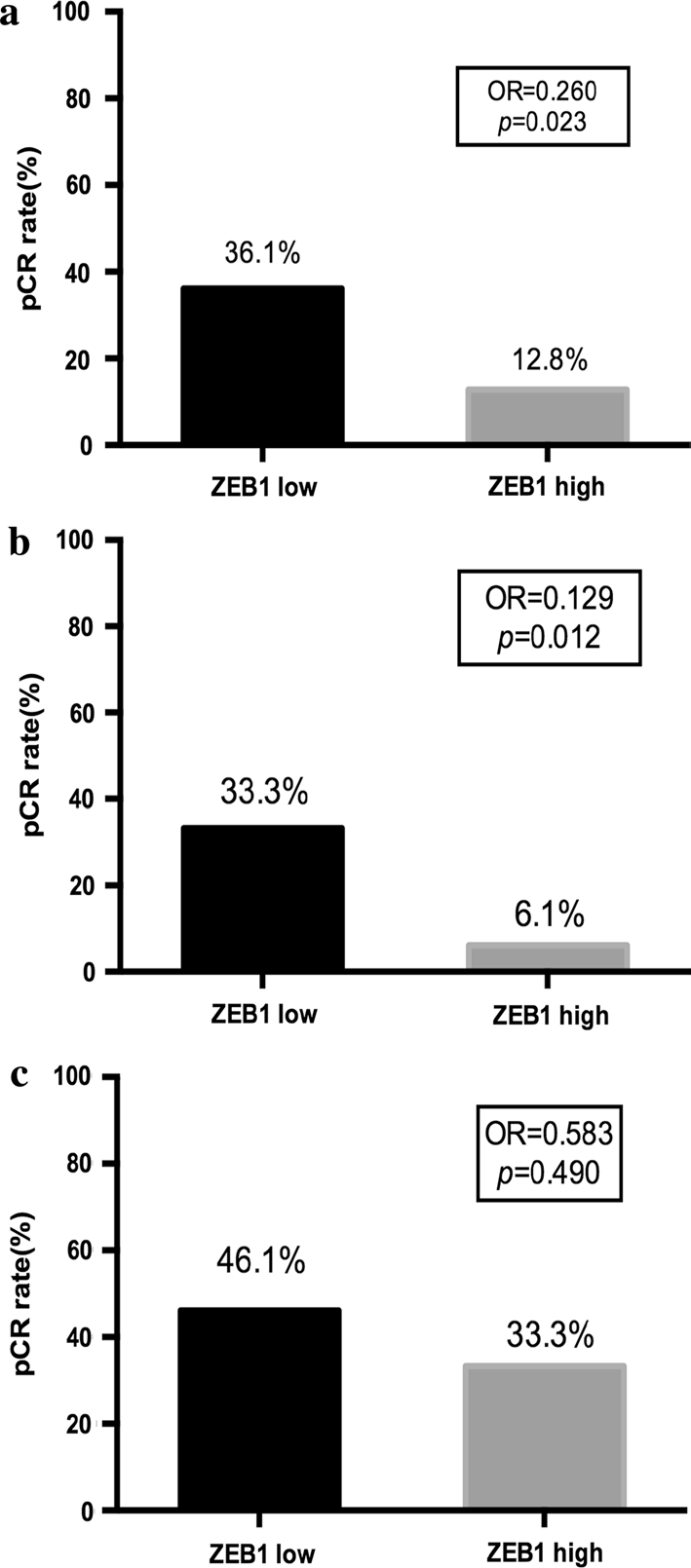
Pathological complete response rates of different ZEB1 expression. **a** Pathological complete response rate of different ZEB1 expression in all patients; **b** Pathological complete response rate of different ZEB1 expression in hormone receptor positive patients; **c** Pathological complete response rate of different ZEB1 expression in HER2 positive patients

The pCR rate of patients with high ZEB1 expression was 12.8%, whereas the pCR rate of patients with negative and low ZEB1 expression was 36.1% (Fig. 2). Univariate logistic regression (OR = 0.260, 95% CI 0.082–0.829, *p *= 0.023) and multivariate logistic regression (OR = 0.074, 95% CI 0.011–0.475, *p *= 0.006) showed that low expression of ZEB1 was an independent predictive factor of the pCR result after NAT. Furthermore, *HER2* amplification (OR = 8.915, 95% CI 1.596–49.811, *p *= 0.013) and high Ki-67 proliferation (OR = 17.138, 95% CI 2.476–118.605, *p *= 0.004) were also predictive factors of pCR (Tables 2, 3).

**Table 2:** Univariate analyses of the predictive markers of pCR

Variables	Comparison for risk ratio	OR	95% confidence interval	*p* value
ZEB1	High expression versus low expression	0.260	0.082–0.829	*0.023*
Age	> 50 versus ≤ 50	1.295	0.446–3.755	0.635
Primary tumor size	> 5 cm versus ≤ 5 cm	2.160	0.731–6.386	0.164
ER status	Positive versus negative	0.188	0.058–0.602	*0.005*
PR status	Positive versus negative	0.306	0.081–1.161	0.082
HER2 status	Positive versus negative	3.697	1.225–11.158	*0.020*
Ki-67	> 30% versus ≤ 30%	6.875	1.789–26.427	*0.005*

Siginificant *p* values are given in italic (*p* < 0.05)

**Table 3:** Multivariate analyses of the predictive markers of pCR

Variables	Comparison for risk ratio	OR	95% confidence interval	*p* value
ZEB1	High expression versus low expression	0.074	0.011–0.475	*0.006*
Age	> 50 versus ≤ 50	1.356	0.318–5.909	0.671
Primary tumor size	> 5 cm versus ≤ 5 cm	2.003	0.498–8.055	0.328
ER status	Positive versus negative	0.468	0.081–2.693	0.395
PR status	Positive versus negative	0.589	0.073–4.718	0.618
HER2 status	Positive versus negative	8.915	1.596–49.811	*0.013*
Ki-67	> 30% versus ≤ 30%	17.138	2.476–118.605	*0.004*

Siginificant *p* values are given in italic (*p* < 0.05)

In the subgroup analysis, a similar trend was observed in HorR-positive patients. The pCR rate of patients with high ZEB1 expression was 6.1%, whereas the pCR rate of patients with negative and low ZEB1 expression was 33.3% (Fig. 2b). Univariate (OR = 0.129, 95% CI 0.026–0.641, *p *= 0.012) and multivariate (OR = 0.054, 95% CI 0.007–0.422, *p *= 0.005) logistic regression tests showed that low ZEB1 expression was associated with a better pCR rate (Tables 4, 5).

**Table 4:** Univariate analyses of the predictive markers of pCR in HorR-positive patients

Variables	Comparison for risk ratio	OR	95% confidence interval	*p* value
ZEB1	High expression versus low expression	0.129	0.026–0.641	*0.012*
Age	> 50 versus ≤ 50	0.825	0.244–2.785	0.757
Primary tumor size	> 5 cm versus ≤ 5 cm	2.086	0.602–7.228	0.246
HER2 status	Positive versus negative	2.697	0.782–9.305	0.116
Ki-67	> 30% versus ≤ 30%	5.079	1.249–20.653	*0.023*

Siginificant *p* values are given in italic (*p* < 0.05)

**Table 5:** Multivariate analyses of the predictive markers of pCR in HorR-positive patients

Variables	Comparison for risk ratio	OR	95% confidence interval	*p* value
ZEB1	High expression versus low expression	0.054	0.007–0.422	*0.005*
Age	> 50 versus ≤ 50	1.242	0.267–5.770	0.782
Primary tumor size	> 5 cm versus ≤ 5 cm	1.923	0.416–8.966	0.400
HER2 status	Positive versus negative	6.654	1.128–39.266	*0.036*
Ki-67	> 30% versus ≤ 30%	10.661	1.725–65.900	*0.011*

Siginificant *p* values are given in italic (*p* < 0.05)

### ZEB1 expression and DFS

**Fig. 3:**
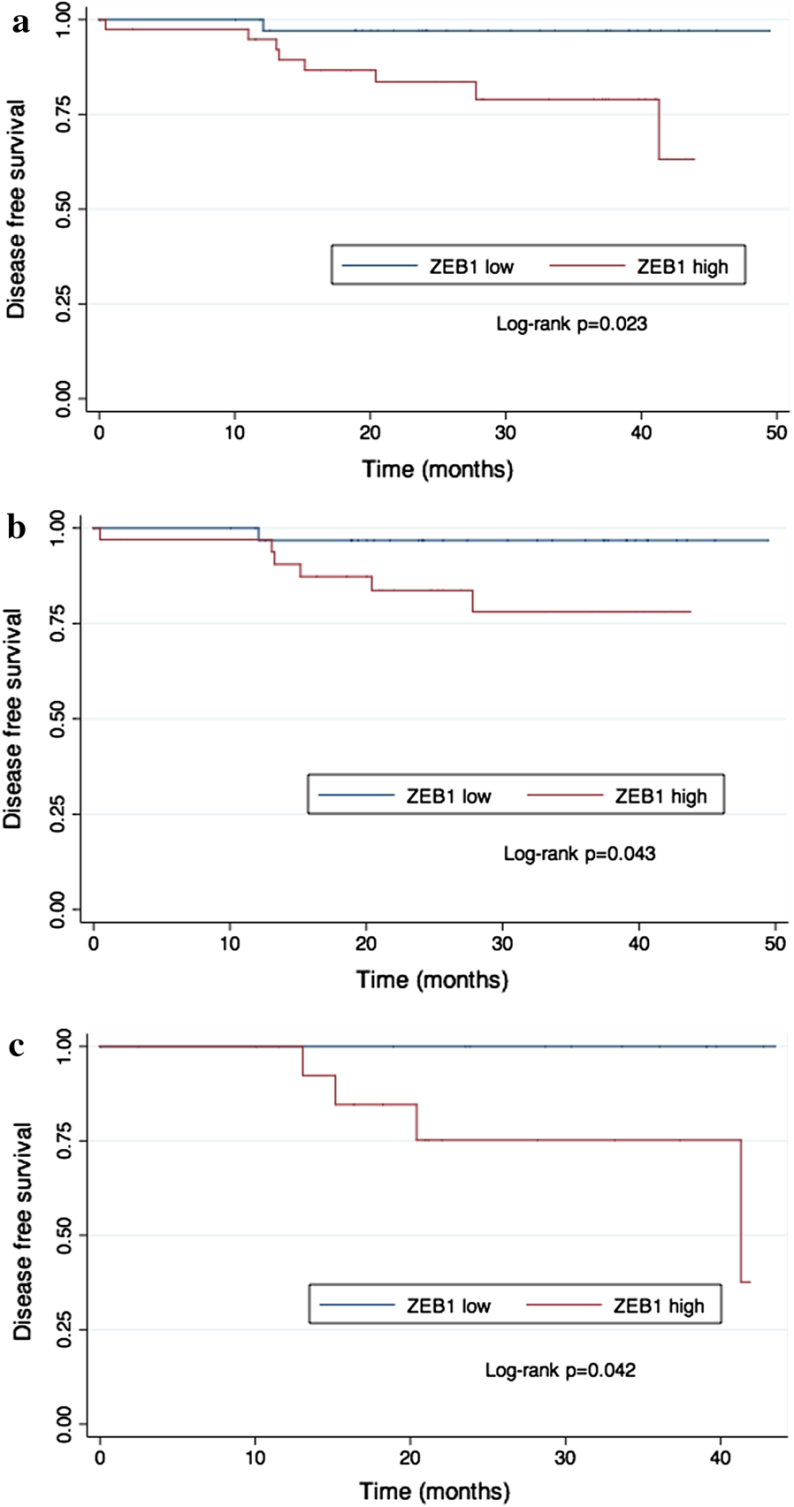
Kaplan–Meier plot of different ZEB1 expression groups. **a** Kaplan–Meier plot of different ZEB1 expression groups in all patients; **b** Kaplan–Meier plot of different ZEB1 expression groups in HorR-positive patients; **c** Kaplan–Meier plot of different ZEB1 expression groups in HER2 positive patients

With a median follow-up time of 27.5 months, patients in the low ZEB1 expression group had significantly fewer disease-free survival events than patients in the high ZEB1 expression group (1 vs 8 events; adjusted HR 9.025, 95% CI 1.024–79.519, *p *= 0.048; Table 6). Kaplan–Meier curves of the two groups separated approximately at 12 months and remained separated for the remainder of the follow-up time (log-rank *p *= 0.023; Fig. 3).

**Table 6:** Cox proportional hazards regression analysis for DFS

Variables	Comparison for risk ratio	HR	95% confidence interval	*p* value
ZEB1	High expression versus low expression	9.025	1.024–79.519	*0.048*
Age	> 50 versus ≤ 50	0.243	0.053–1.098	0.066
Primary tumor size	> 5 cm versus ≤ 5 cm	4.071	0.902–18.384	0.068
ER status	Positive versus negative	1.464	0.089–24.016	0.789
PR status	Positive versus negative	0.082	0.003–2.090	0.130
HER2 status	Positive versus negative	3.280	0.709–15.183	0.129
Lymph node positive	> 0 versus ≤ 0	11.362	1.129–114.303	*0.039*

Siginificant *p* values are given in italic (*p* < 0.05)

In HorR-positive patients, the Kaplan–Meier plot showed that patients with high ZEB1 expression exhibited poorer survival compared with those with low ZEB1 expression (log-rank *p *= 0.043; Fig. 3b). The Cox hazard model also suggested a higher risk of recurrence in the high ZEB1 expression group (HR = 10.516, 95% CI 1.171–94.435, *p *= 0.036; Table 7). In addition, primary tumor size (HR = 11.202, 95% CI 1.203–104.163, *p *= 0.034) was an independent predictor of DFS in HorR-positive patients.

**Table 7:** Cox proportional hazards regression analysis for DFS in HorR-positive patients

Variables	Comparison for risk ratio	HR	95% confidence interval	*p* value
ZEB1	High expression versus low expression	10.516	1.171–94.435	*0.036*
Age	> 50 versus ≤ 50	0.445	0.088–2.262	0.329
Primary tumor size	> 5 cm versus ≤ 5 cm	11.202	1.203–104.163	*0.034*
HER2 status	Positive versus negative	1.129	0.212–6.015	0.887
Lymph node positive	> 0 versus ≤ 0	2.762	0.457–16.685	0.268

Siginificant *p* values are given in italic (*p* < 0.05)

HER2-positive patients in the low ZEB1 expression group had significantly fewer survival events than patients in the high ZEB1 expression group (0 versus 4 events; log-rank *p *= 0.042; Fig. 3c). However, this result was not observed in the Cox regression analysis (data not shown).

**Fig. 4:**
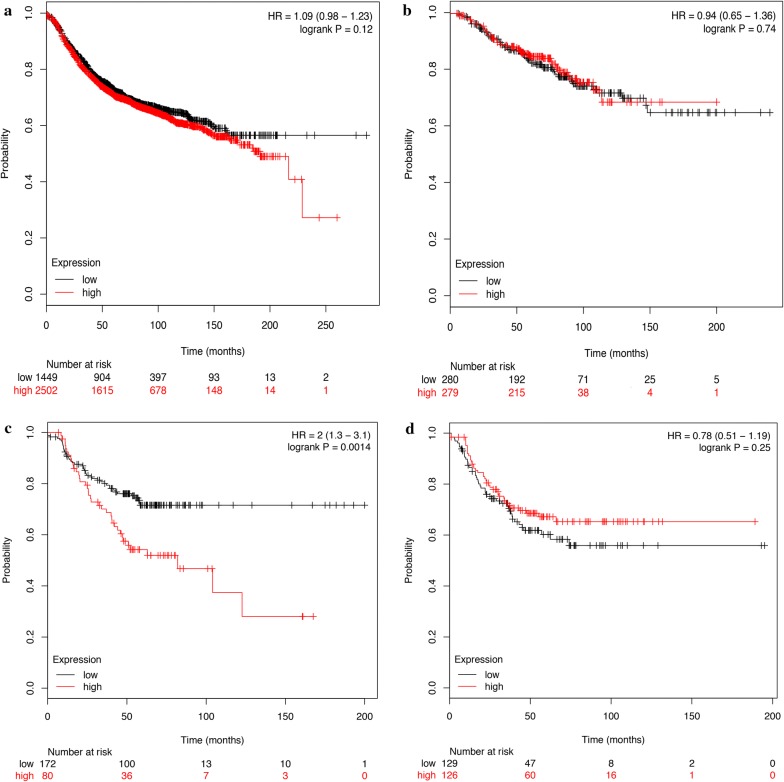
Kaplan–Meier plot of different *ZEB1* mRNA expression groups (KMPlotter). **a** Kaplan–Meier plot of different ZEB1 expression groups in all patients; **b** Kaplan–Meier plot of different ZEB1 expression groups in HorR-positive patients; **c** Kaplan–Meier plot of different ZEB1 expression groups in HER2 positive patients; **d** Kaplan–Meier plot of different ZEB1 expression groups in TNBC patients

We failed to perform an effective subgroup analysis due to the small number of patients with TNBC breast cancer. Considering the relatively small sample size of our study, we searched an online database to validate our results. We used KM plotter (http://kmplot.com/analysis/) to draw survival curves of patients with different *ZEB1* mRNA levels detected by mRNA gene chip [20]. In the total patient cohort, the HorR-positive subgroup and the TNBC subgroup, *ZEB1* mRNA levels were not significantly correlated with relapse-free survival (RFS). In the HER2-overexpressing subgroup, patients with high *ZEB1* mRNA levels were more likely to suffer from disease relapse (Fig. 4).

## Discussion

Our research demonstrated that ZEB1 expression is an independent negative predictive factor of pCR and a prognostic biomarker of DFS in HorR-positive and HER2 overexpressing breast cancer patients. To the best of our knowledge, this is the first time that the predictive value of ZEB1 in NAT has been indicated for breast cancer patients.

ZEB1 is usually involved in EMT and tumor metastasis [10]. Previous studies have reported its indication of unfavorable clinical factors, such as larger tumor size, more lymph node metastasis and higher tumor stage, in breast cancer [21, 22]. However, ZEB1 expression was only related to patient age and menstrual status in our study. The patients included in our study were from NAT clinical trials. We enrolled patients with locally advanced breast cancer. Most patients exhibited large tumor size and high Ki-67 levels, which were consistent with the features of locally advanced breast cancer. This might explain why ZEB1 expression was not relevant to tumor size, Ki-67 level or other clinical factors in our study. On the other hand, the age and menopausal status of the patients were irrelevant to the disease at enrollment, which might reflect the characteristics of ZEB1-positive tumors.

For the first time, we revealed a negative correlation between ZEB1 expression and pCR in breast cancer patients receiving NAT. We found that tumors expressing high levels of ZEB1 protein were less likely to achieve pCR after NAT. In the preclinical stage, a study from Zhang reported that ZEB1 was correlated with several chemo-resistant genes, such as *ATM*, *CD4*, and *PIM3* [10]. ZEB1 expression was associated with a chemo-resistant tumor phenotype both in vitro and in vivo. Others also reported that ZEB1 promoted the DNA damage response and radio-resistance through CHK1 [23]. All this molecular evidence suggested that ZEB1 was able to undermine the sensitivity of tumors towards cytotoxic therapy, which could explain why it was more difficult for patients with high ZEB1 expression levels to achieve complete remission after NAT compared with patients with low ZEB1 expression levels. Moreover, ZEB1 expression was still a strong indicator of poor survival in breast cancer patients in our study. The fact that ZEB1 promoted EMT and facilitated tumor metastasis is probably the main underlying reason for this result. Our result is supported by research by Lin, which showed that *ZEB1* and *ZEB2* mRNA expression as well as protein expression were associated with poor survival in breast cancer [21]. Our result was also supported by Min, who reported that ZEB1 protein expression was associated with poor survival in TNBC [24]. The predictive role of ZEB1 is also validated in other tumors, such as ovarian carcinoma and hepatocellular carcinoma [25–27].

In subgroup analyses, we found a similar trend in HorR-positive patients both in pCR and DFS. Though not observed in our study, it was reported that *ZEB1* gene expression was more frequent in hormone-positive tumors [28]. To date, an ideal biomarker to predict pCR in HorR-positive patients remains to be identified. According to our results, ZEB1 expression might be used as a potential predictive biomarker of pCR in HorR-positive patients.

There are several deficits in our study. Due to the short starting time of our two clinical trials, there were few patients enrolled in the study, resulting in the failure to perform subgroup analyses in TNBC. In addition, due to the short follow-up period, we were not able to perform an overall survival (OS) analysis, which is pending further follow-ups and analyses. To supplement our study, we analyzed the online database KM plotter with larger patient data. Using mRNA gene CHIP detection, *ZEB1* mRNA expression was a poor prognostic factor in the HER2-overexpressing subgroup. However, in all patients as well as HorR-positive and TNBC patients, *ZEB1* expression lost its predictive value. The discordance may be explained by posttranscriptional editing. Llorens demonstrated that ZEB1 activity was dependent on its phosphorylation status [29]. Thus, the predictive value of *ZEB1* mRNA level might be different from ZEB1 protein expression.

## Conclusions

In conclusion, our results showed that high ZEB1 protein expression was a negative predictive marker of pCR and DFS in neoadjuvant therapy in breast cancer patients and in HorR-positive and HER2-overexpressing subgroups.

## Abbreviations

ZEB1: zinc finger E-box binding homeobox 1
EMT: epithelial-to-mesenchymal transition
IHC: immunohistochemistry
NAT: neoadjuvant therapy
pCR: pathological complete response
DFS: disease-free survival
HR: hazard ratio
95% CI: 95% confidential interval
ER: estrogen receptor
PR: progesterone receptor
OR: odds ratio
HorR-positive: hormone receptor-positive
RFS: relapse-free survival
OS: overall survival
